# Liraglutide prevents cellular senescence in human retinal endothelial cells (HRECs) mediated by SIRT1: an implication in diabetes retinopathy

**DOI:** 10.1007/s13577-024-01038-1

**Published:** 2024-03-04

**Authors:** Lihua Hou, Jianying Du, Yongxiao Dong, Min Wang, Libo Wang, Jifei Zhao

**Affiliations:** 1 Department of Ophthalmology, The First People’s Hospital of Xianyang, No. 10, Biyuan Road, Qindu District, Xianyang City, 712000 Shanxi China; 2 Department of Ophthalmology, Sanyuan Eye Hospital, Xianyang City, 713899 Shanxi China

**Keywords:** Diabetic retinopathy, Glucagon-like peptide 1 (GLP-1), Liraglutide, Senescence, High glucose

## Abstract

Diabetes mellitus (DM) is a chronic metabolic disorder affecting millions of people worldwide, characterized by dysregulated glucose homeostasis and hyperglycemia. Diabetic retinopathy (DR) is one of the serious multisystemic complications. Aging is an important risk factor for DR. Endothelial sirtuin 1 (SIRT1) plays an important role in regulating the pathophysiology of glucose metabolism, cellular senescence, and aging. Liraglutide, an analog of Glucagon-like peptide 1 (GLP-1), has been widely used in the treatment of DM. However, the effects of Liraglutide on DR are less reported. Here, we investigated whether treatment with Liraglutide has beneficial effects on high glucose (HG)-induced injury in human retinal microvascular endothelial cells (HRECs). First, we found that exposure to HG reduced the expression of glucagon-like peptide 1 receptor 1 (GLP-1R). Additionally, Liraglutide ameliorated HG-induced increase in the expression of vascular endothelial growth factor-A (VEGF-A) and interleukin 6 (IL-6). Importantly, Liraglutide ameliorated cellular senescence and increased telomerase activity in HG-challenged HRECs. Liraglutide also reduced the levels of p53 and p21. Mechanistically, Liraglutide restored the expression of SIRT1 against HG. In contrast, the knockdown of SIRT1 abolished the protective effects of Liraglutide in cellular senescence of HRECs. Our findings suggest that Liraglutide might possess a benefit on DR mediated by SIRT1.

## Introduction

Diabetes mellitus (DM) is one of the chronic diseases that seriously jeopardizes human health. The prevalence of DM in China is approximately 11.6% [1]. Diabetic retinopathy (DR) is the most common microvascular complication of diabetes and the leading cause of preventable blindness in the working-age population [2]. At present, nearly 100 million patients worldwide suffer from DR, and with the increase in the prevalence of diabetes, this number will continue to increase, becoming a huge burden on social health [3]. Clinically, DR can be divided into non-proliferative DR (NPDR) and proliferative DR (PDR) according to the degree of lesions [4]. Currently, the treatment methods for DR are limited, and strict blood glucose control of retinal photocoagulation is an effective means to prevent and treat DR. However, peripheral visual field defects will be induced by the invasive nature of retinal photocoagulation [5, 6]. Intravitreal injection of anti-vascular endothelial growth factor (VEGF) shows a definite curative effect on macular edema caused by vascular leakage. However, the treatment is expensive and requires repeated injections, which fail in reversing the lost vision [7, 8]. Therefore, it is an urgent task to develop novel effective, precise, and non-invasive treatments for clinical DR. The pathogenesis of DR is complex. Endothelial cell damage induced by hyperglycemia contributes to the enlargement of the permeability of retinal capillaries, which is an important inducer of microvascular lesions [9]. However, the exact pathway by which hyperglycemia induces endothelial cell damage is still unclear, and the main theories include oxidative stress, polyol pathway, advanced glycation end products (AGEs), and inflammatory response [10]. Aging is an independent risk factor for cardiovascular disease. Senescent vascular cells can be found in human atherosclerotic tissues, suggesting that the pathophysiological basis of age-related vascular diseases may be due to the senescence of vascular cells [11]. Senescence of vascular cells is also observed in diabetes-related vascular lesions [12]. High glucose induces senescence of endothelial cells, leads to vascular inflammation and blood embolism, and further contributes to the development of diabetic cardiovascular diseases. The risk of age-related diseases, such as cardiovascular disease, can be alleviated by caloric restriction [13]. Malika Oubaha claimed that the pathological angiogenesis in DR resulted from senescence-associated secretory phenotype [14]. Therefore, cellular senescence will be a promising target for DR treatment.

Liraglutide is an agonist of GLP-1R, which is primarily used for the treatment of type 2 diabetes mellitus, and administered by subcutaneous injection once daily [15]. Liraglutide inhibits glucagon secretion in a glucose-dependent mode, promotes insulin secretion, and reduces blood glucose, so the risk of hypoglycemia is very low [15]. It has also been reported to delay gastric emptying [16], inhibit glucagon secretion [17], reduce appetite, and suppress weight gain [18]. Furthermore, insulin resistance (IR) can be improved by Liraglutide by promoting the regeneration and differentiation of pancreatic β-cells, reducing the death of β-cells, and accelerating fat decomposition [19]. Promising therapeutic effects against diabetes have been achieved by Liraglutide in several reports [20]. The present study will explore the potential application of Liraglutide in the treatment of DR by studying the impact of Liraglutide on HG-induced cellular senescence in human retinal microvascular endothelial cells (HRECs).

## Materials and methods

### Cell culture, treatment, lentiviral SIRT1 shRNA preparation and transduction

HRECs [21] were obtained from Shanghai Xuanke Biotechnology co. LTD (Shanghai, China) and cultured in endothelial cell medium (Sciencell, USA) containing 10% fetal bovine serum (FBS) (Gibco, USA). Cells were incubated at 37 ℃ under 5% CO_2_. Unless specially explained, the cells were used after they reached 85% confluence at passages 3–6.

The chemically synthesized sense (5′-TGCGGGAATCCAAAGGATAATTTTCAAGAGAAATTATCCTTTGGATTCCCGCTTTTTC-3′) and antisense (5′-TCGAGAAAAAGCGGGAATCCAAAGGAGAAGGTCTCTTGAAAATTATCCTTTGGATTCCCGA-3′) chains were annealed to form SIRT1 shRNA expression templates, which were then connected to the vector pLentilox3.7 containing green fluorescent protein (GFP). The obtained plasmids were cotransfected with lentiviral packaging plasmids pLP1, pLP2, and pLP/VSVG into 293FT cells. After 24 h of transfection, the culture medium was changed. After 48 h of cultivation, the cell supernatant rich in lentivirus particles was collected and concentrated 20 times to obtain a high titer lentivirus concentrate.

To knockdown the expression of SIRT1 in HRECs, cells were transduced with lentiviral SIRT1 shRNA for 48 h, followed by evaluating the transduction efficacy using the Western blotting assay.

### Lactate dehydrogenase (LDH) release

In a 96-well plate, cell supernatant was collected and implanted, followed by adding LDH solution to be incubated for 90 min in the dark. Subsequently, the absorbance at 490 nm was detected utilizing the microplate reader (PerkinElmer, USA). The release of LDH was calculated based on the absorbance value of the testing sample and standards.

### 3-(4,5-Dimethylthiazol-2-yl)-2,5-diphenyltetrazolium bromide (MTT) assay

HRECs were implanted in a 96-well plate and incubated at 37 ℃ for 1 day. Different concentrations of Liraglutide (5, 10, 50, 100, 500, 1000 nM) were introduced, incubated for 1 day, and then 10 μL of MTT (Sigma-Aldrich, USA) solution was added. Lastly, the microplate reader (PerkinElmer, USA) was utilized to detect the OD value at 490 nm [22].

### Real-time polymerase chain reaction (PCR)

RNAs were isolated from treated HRECs with the TRIzol solution and centrifuged at 16,000 *g* for 10 min, followed by being dissolved in ddH_2_O. The transcription to cDNA was conducted using a reverse transcription kit (QIAGEN, Germany). The PCR was then carried out using the ABI 7500 Real-time PCR system (Applied Biosystems, USA) and the SYBR green (Sigma, USA). After normalization with glyceraldehyde-3-phosphate dehydrogenase (GAPDH), the level of genes was calculated with the 2^−ΔΔCt^ method. The following primers were used in this study: GLP-1R (F, 5′-CTACGCACTCTCCTTCTCTGCT-3′; R, 5′- CGGACAATGCTCGCAGGATGAA-3′), p53 (F, 5′-CCTCAGCATCTTATCCGAGTGG-3′; R, 5′-TGGATGGTGGTACAGTCAGAGC-3′), p21 (F, 5′'-AGGTGGACCTGGAGACTCTCAG-3′; R, 5′-TCCTCTTGGAGAAGATCAGCCG-3′), SIRT-1 (F, 5′-TAGACACGCTGGAACAGGTTGC-3′; R, 5′-CTCCTCGTACAGCTTCACAGTC-3′), GAPDH (F, 5′-GTCTCCTCTGACTTCAACAGCG-3′; R, 5′-ACCACCCTGTTGCTGTAGCCAA-3′).

### Western blotting assay

The bicinchoninic acid (BCA) method was used to measure the concentration of proteins after they had been isolated from HRECs using various treatment methods before being further separated using 12% SDS-PAGE. Then, proteins were transferred onto the PVDF membrane, which was blocked with skim milk for 2 h. Subsequently, the membrane was incubated with primary antibodies against GLP-1R (1:800; cat. no. sc-390774, Santa Cruz Biotechnology, USA), p53 (1:2000; cat. no. sc-126, Santa Cruz Biotechnology, USA), p21 (1:2000; cat. no. sc-6246, Santa Cruz Biotechnology, USA), SIRT1 (1:3000, cat. no. sc-74465, Santa Cruz Biotechnology, USA), or GAPDH (1:5000, cat. no. sc-365062, Santa Cruz Biotechnology, USA) for 12 h at 4 ℃, followed by adding the secondary antibody (1:2000, cat. no. 7074 or cat. no.7076, Cell Signaling Technology, USA) for 1.5 h. Lastly, Bio-Rad Quantity One software was used for the quantitative analysis of bands.

### Enzyme-linked immunosorbent assay (ELISA)

The precipitates were discarded after centrifugation at 1000 × *g* for 10 min at 4 °C, and then the standard dilution and test samples were added to the 96-well plate. After introducing the biotin-labeled antibody, samples were incubated at 37 ℃ for 1 h and the liquid was then discarded, followed by adding the HRP solution. After incubation at 37 ℃ for half an hour, substrate A and substrate B were introduced into each well and incubated at 37 ℃ for 15 min, and the stop solution was added subsequently. Finally, the microplate reader (PerkinElmer, USA) was utilized to detect the optical density (OD) value at 450 nm.

### Senescence-associated β-galactosidase (SA-β-gal) staining

HRECs were washed with the phosphate-buffered saline (PBS) buffer and treated with 1 mL fixing solution for 15 min, followed by being rinsed with 2 mL PBS buffer. Then, cells were incubated with 1 mL SA-β-gal staining solution and the culture plate was cultured at 37 ℃ without CO_2_ for 15 h. After sealing, the number of positive cells was counted using a microscope (Leica, Germany) [23].

### The telomerase activity

HRECs were lysed with the CHAPS buffer and the sample was centrifuged at 15,000 × *g* for one and a half hours, followed by being quantified with the BCA method. The activity of telomerase was then determined utilizing the TeloTAGGG Telomerase PCR ELISA Plus kit (Roche, Switzerland) based on the instruction of the product. The TRAP-PCR system, the samples, and the primers were introduced together, followed by conducting the quantification utilizing the RT-PCR assay.

### Statistical analysis

Achieved data were expressed as mean ± standard deviation (S.D.) and were analyzed with the GraphPad software. The analysis of variance (ANOVA) method with Tukey’s post hoc test was applied for comparison. *P* < 0.05 was regarded as a significant difference.

## Results

### High glucose reduced the expression of GLP-1R in HRECs

**Fig. 1:**
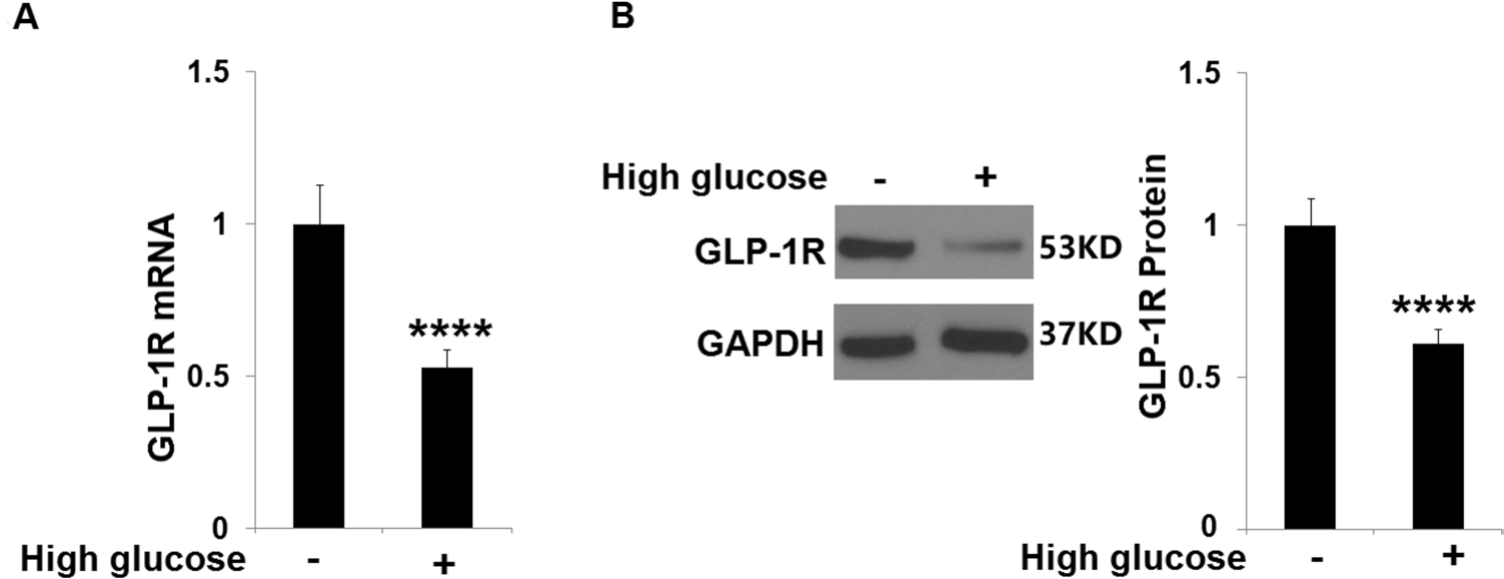
High glucose reduced the expression of GLP-1R in HRECs. Cells were stimulated with normal glucose (5 mM) or high glucose (30 mM) for 24 h. (**A**). mRNA levels of GLP-1R; (**B**). Protein levels of GLP-1R (*n* = 6 ****, *P* < 0.0001 vs. vehicle group)

To examine the potential effect of Liraglutide on HG-trigged damage in HRECs, cells were stimulated with normal glucose (5 mM) or HG (30 mM) for 24 h, followed by detecting the expression level of GLP-1R. We found that GLP-1R (Fig. 1) was extremely downregulated in HG-stimulated HRECs, suggesting an inactivated GLP-1R signal in HG-stimulated HRECs.

### The cytotoxicity of Liraglutide in HRECs

**Fig. 2:**
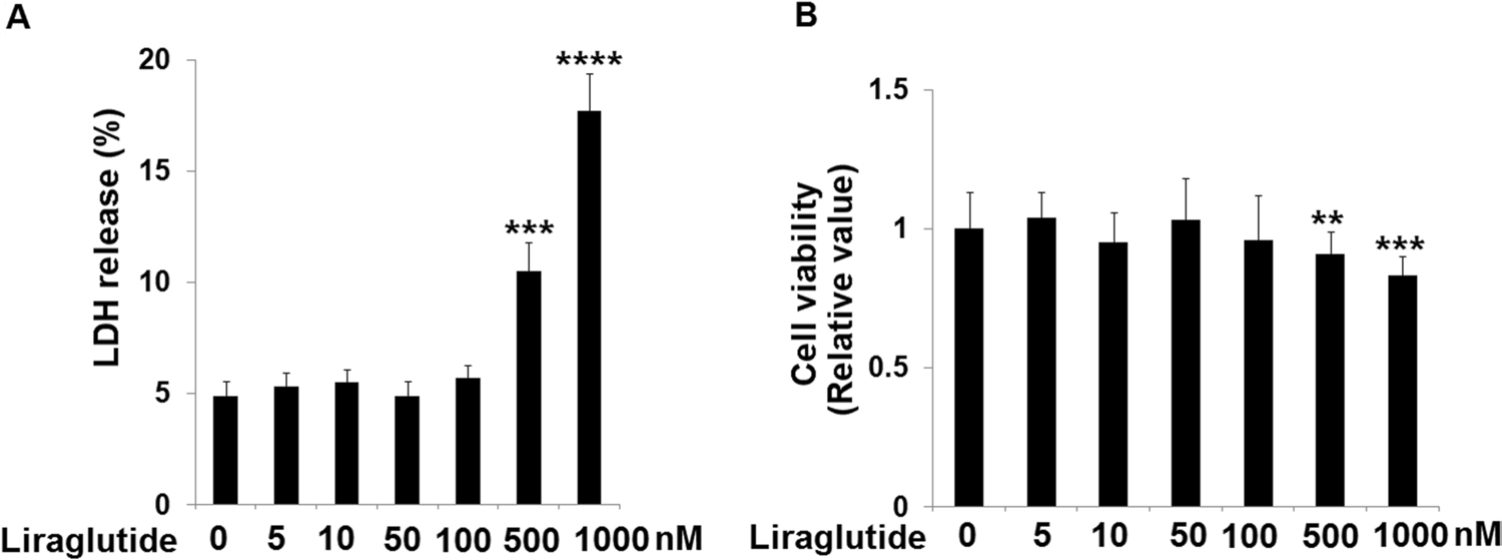
The cytotoxicity of Liraglutide in HRECs. Cells were stimulated with 0, 5, 10, 50, 100, 500, 1000 nM Liraglutide for 24 h. (**A**). LDH release; (**B**). Cell viability (n = 6 **, ***, ****, *P* < 0.01, 0.001, 0.0001 vs. vehicle group)

HRECs were stimulated with 5, 10, 50, 100, 500, 1000 nM Liraglutide for 24 h, followed by detecting the LDH release and cell viability to determine the optimized Liraglutide concentration. The LDH release was maintained at around 5% (Fig. 2A) as the concentration of Liraglutide was promoted from 5 to 100 nM, which was greatly increased to 10.5 and 17.7% under the stimulation of 50 and 100 nM Liraglutide. Moreover, the cell viability (Fig. 2B) was slightly changed as the concentration of Liraglutide was promoted from 5 to 100 nM and dramatically declined in the 500 and 1000 nM Liraglutide groups. Therefore, we chose 50 and 100 nM as the concentrations applied in in vitro assays.

### Liraglutide reduced the expressions of VEGF-A and IL-6 in HG-stimulated HRECs

**Fig. 3:**
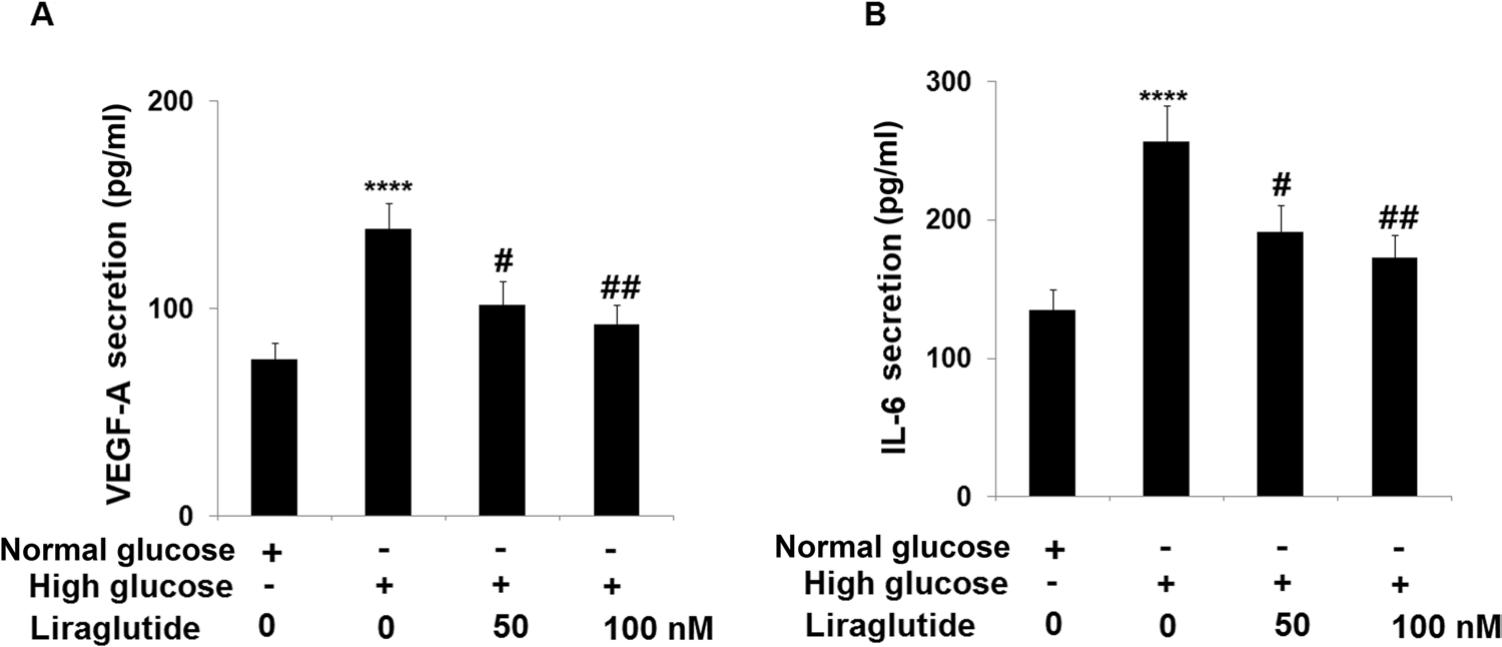
Liraglutide reduced the expression of VEGF-A and IL-6 in high glucose-challenged HRECs. Cells were stimulated with high glucose (30 mM) with or without Liraglutide (50, 100 nM) for 24 h. (**A**). The levels of VEGF-A; (**B**). The levels of IL-6 (*n* = 6 ****, *P* < 0.0001 vs. vehicle group; #, ##, *P* < 0.05, 0.01 vs. high glucose group)

The secretion of VEGF and IL-6 plays a critical role in diabetic eye diseases [18, 19]. Cells were treated with 30 mM glucose with or without Liraglutide (50, 100 nM) for 24 h, followed by determining the level of VEGF-A and IL-6 by ELISA. The level of VEGF-A (Fig. 3A) was found to be dramatically increased from 75.6 to 138.6 pg/mL in HG-stimulated HRECs and reduced to 102.1 and 92.5 pg/mL by 50 and 100 nM Liraglutide, respectively. Furthermore, the section of IL-6 (Fig. 3B) in the control, HG, 50 nM Liraglutide, and 100 nM Liraglutide groups was 135.2, 256.8, 191.6, and 172.9 pg/mL, respectively. These results suggested that the release of VEGF-A and IL-6 in HG-stimulated HRECs was repressed by Liraglutide.

### Liraglutide alleviated cellular senescence in HG-stimulated HRECs

**Fig. 4:**
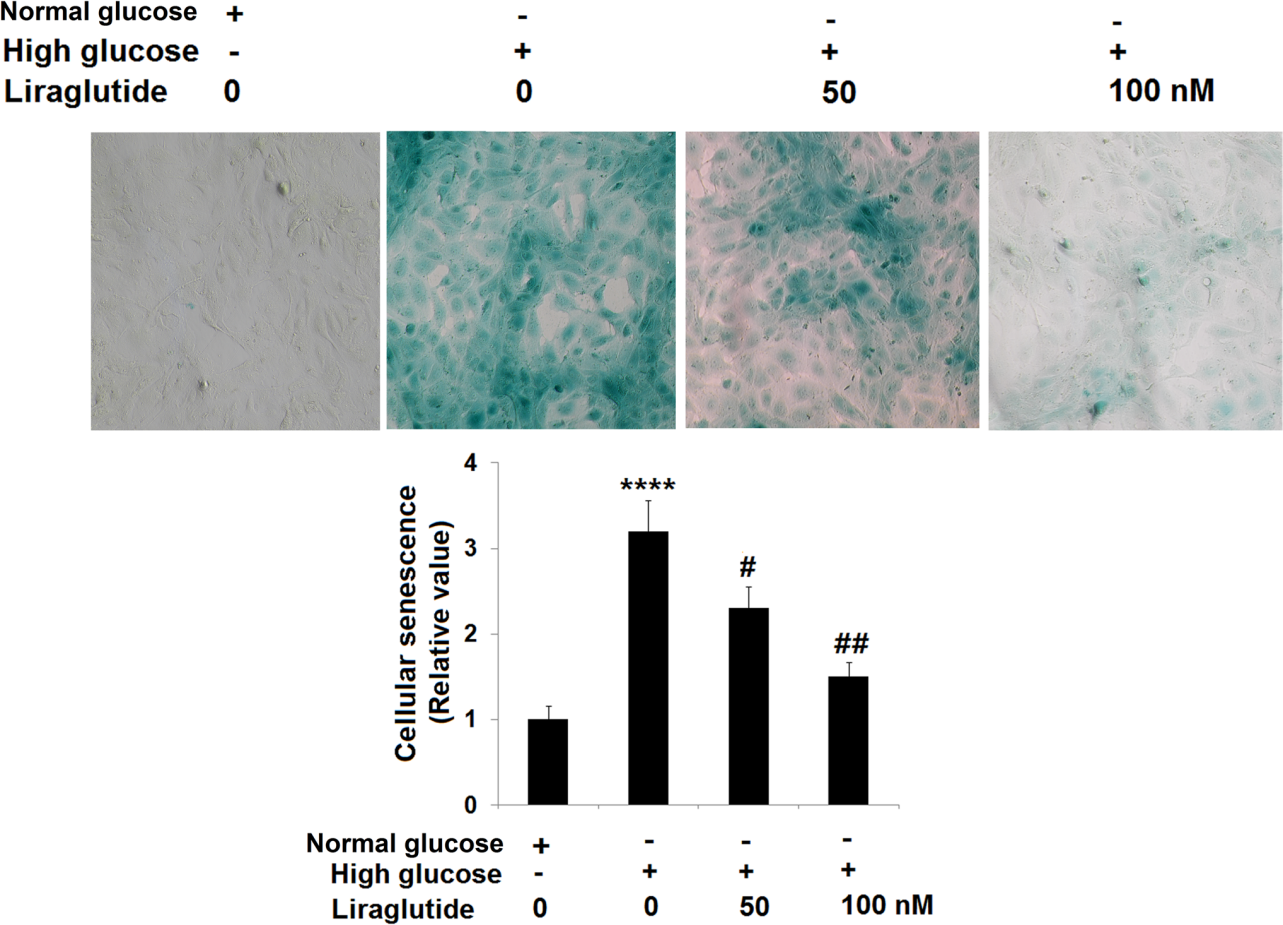
Liraglutide ameliorated cellular senescence in high glucose-challenged HRECs. Cells were stimulated with high glucose (30 mM) with or without Liraglutide (50, 100 nM) for 14 days. Cellular senescence was measured using SA-β-gal staining (*n* = 5 ****, *P* < 0.0001 vs. vehicle group; #, ##, *P* < 0.05, 0.01 vs. high glucose group)

HRECs were treated with 30 mM glucose with or without Liraglutide (50, 100 nM) for 10 days, followed by evaluating the state of cellular senescence. We found that the percentage of SA-β-gal staining positive cells (Fig. 4) was extremely increased in HG-stimulated HRECs and greatly repressed by 50 and 100 nM Liraglutide, suggesting an inhibitory effect of Liraglutide on cellular senescence in HG-stimulated HRECs.

### Liraglutide promoted telomerase activity in HG-stimulated HRECs

**Fig. 5:**
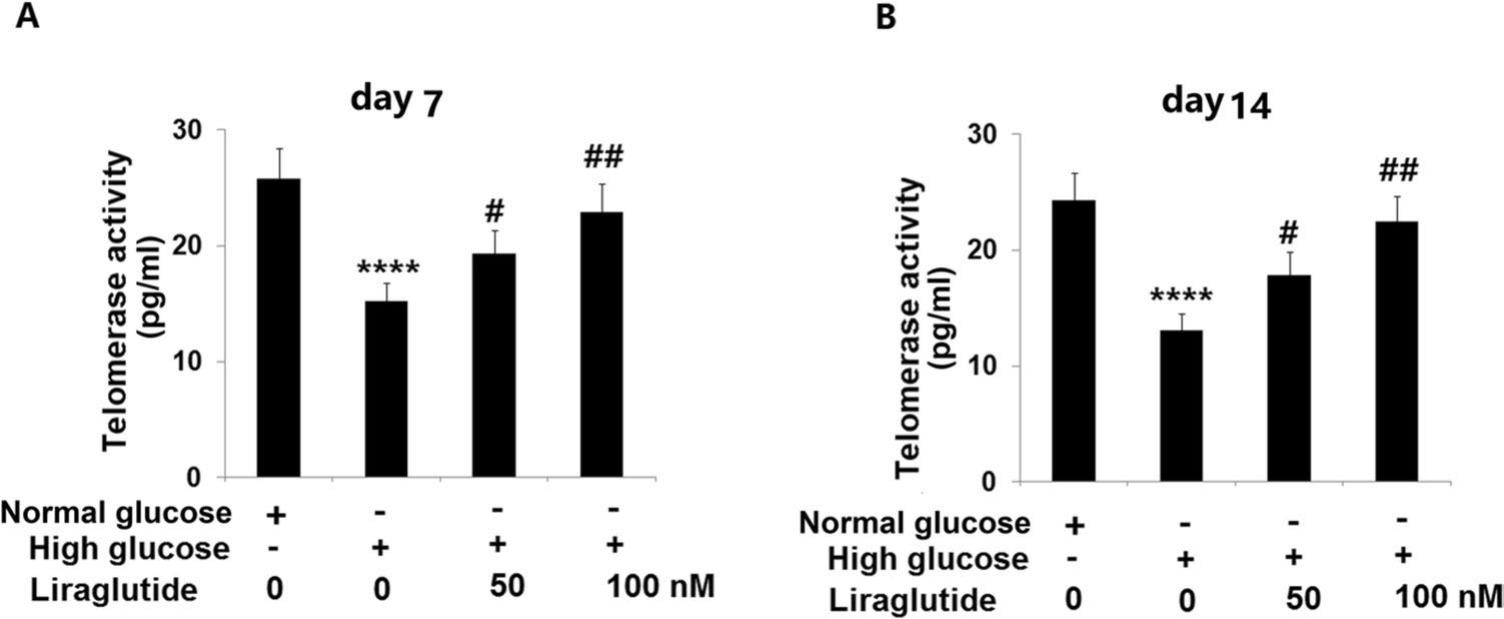
Liraglutide increased telomerase activity in high glucose-challenged HRECs. (**A**) Telomerase activity in 7 days; (**B**) Telomerase activity in 14 days (*n* = 5, ****, *P* < 0.0001 vs. vehicle group; #, ##, *P* < 0.05, 0.01 vs. high glucose group)

The telomerase activity in HRECs was further assessed for 7 days and 14 days after treatments. In 7 days, the telomerase activity in HRECs (Fig. 5A) declined from 25.8 to 15.2 IU/L by 30 mM glucose and elevated to 19.3 and 22.9 IU/L by 50 and 100 nM Liraglutide, respectively. Furthermore, in 14 days, the telomerase activity (Fig. 5B) in the control, HG, 50 nM Liraglutide, and 100 nM Liraglutide groups was 24.3, 13.1, 17.8, and 22.5 IU/L, respectively. These data further confirmed the impact of Liraglutide on cellular senescence in HG-stimulated HRECs.

### Liraglutide reduced the levels of p53 and p21 in HG-challenged HRECs

**Fig. 6:**
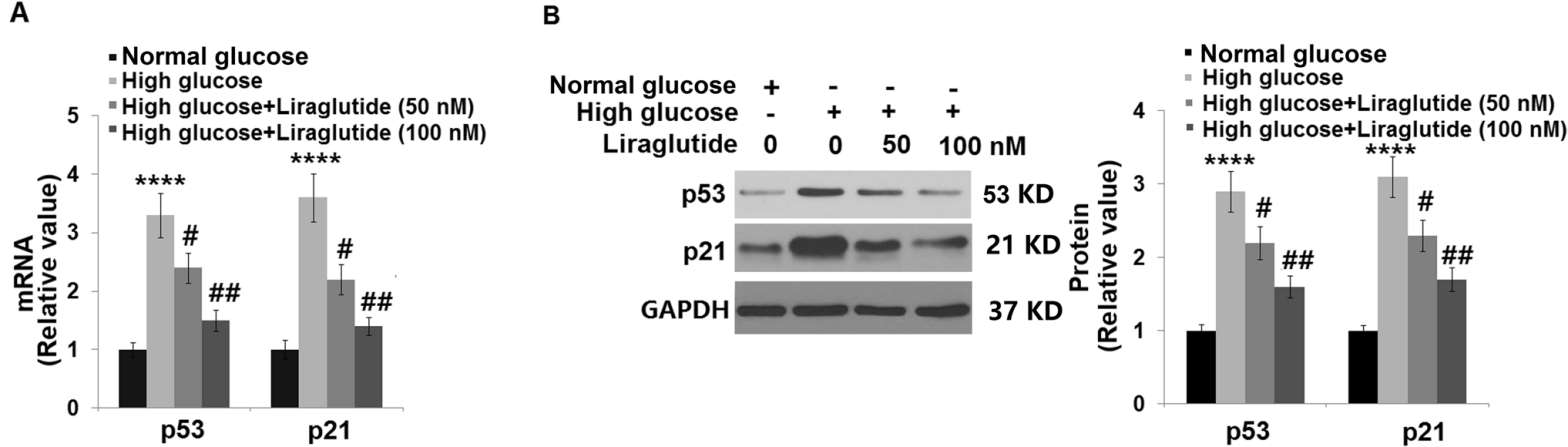
Liraglutide reduced the levels of p53 and p21 in high glucose-challenged HRECs. Cells were stimulated with high glucose (30 mM) with or without Liraglutide (50, 100 nM). (**A**) mRNA of p53 and p21; (**B**) Protein of p53 and p21 (*n* = 5, ****, *P* < 0.0001 vs. vehicle group; #, ##, *P* < 0.05, 0.01 vs. high glucose group)

Subsequently, the level of biomarkers of cellular senescence was examined. We found that in HG-stimulated HRECs, p53 and p21 (Fig. 6) were dramatically upregulated and the expression level was greatly repressed by 50 and 100 nM Liraglutide, indicating an inhibitory property of Liraglutide against p53/p21 axis in HG- challenged HRECs.

### Liraglutide restored the expression of SIRT1 in HG-stimulated HRECs

**Fig. 7:**
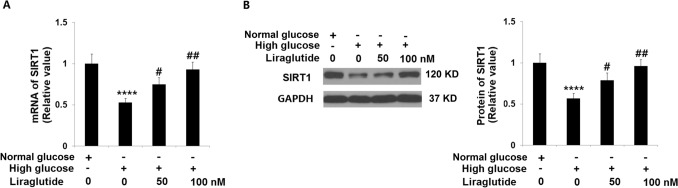
Liraglutide restored the expression of SIRT1 in high glucose-challenged HRECs. Cells were stimulated with high glucose (30 mM) with or without Liraglutide (50, 100 nM). (**A**) mRNA of SIRT1; (**B**) Protein of SIRT1 (*n* = 5, 6, ****, *P* < 0.0001 vs. vehicle group; #, ##, *P* < 0.05, 0.01 vs. high glucose group)

SIRT1 is an important regulator in the progression of cellular senescence [24]. In HG-stimulated HRECs, the level of SIRT1 (Fig. 7) was extremely declined and greatly rescued by 50 and 100 nM Liraglutide, suggesting the regulatory effect of Liraglutide on HG-induced cellular senescence in HRECs might be mediated by SIRT1.

### Knockdown of SIRT1 abolished the protective effects of Liraglutide in cellular senescence against HG

**Fig. 8:**
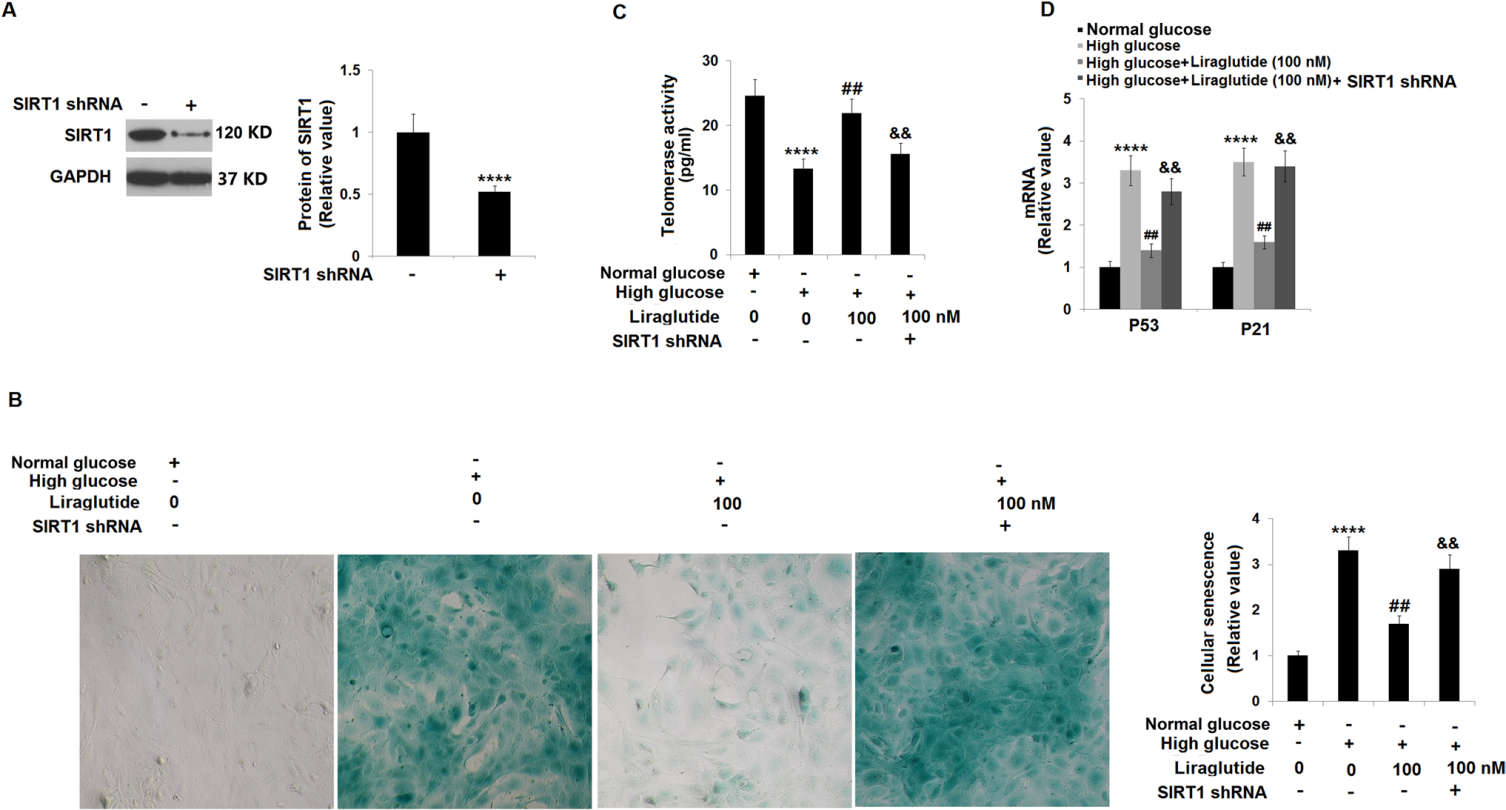
Knockdown of SIRT1 abolished the protective effects of Liraglutide in cellular senescence against high glucose. Cells were transduced with lentiviral SIRT1 shRNA, followed by stimulation with high glucose (30 mM) with or without Liraglutide (100 nM) for 14 days. (**A**) Western blot analysis revealed successful knockdown of SIRT1; (**B**) Cellular senescence was measured using SA-β-gal staining; (**C**) Telomerase activity in 14 days; (**D**) mRNA of p53 and p21 (*n* = 5, ****, *P* < 0.0001 vs. vehicle group; ##, *P* < 0.01 vs. high glucose group; &&, *P* <0.01 vs. high glucose + Liraglutide group)

To verify that SIRT1 mediated the function of Liraglutide, HRECs were transduced with lentiviral SIRT1 shRNA, followed by stimulation with HG (30 mM) with or without Liraglutide (100 nM) for 14 days. The transduction efficacy was identified using a Western blotting assay (Fig. 8A). The proportion of SA-β-gal staining positive cells in HG-stimulated HRECs was greatly declined by 100 nM Liraglutide and dramatically rescued by the knockdown of SIRT1 (Fig. 8B). Furthermore, the telomerase activity (Fig. 8C) in the control, HG, 100 nM Liraglutide, and 100 nM Liraglutide + lentiviral SIRT1 shRNA groups was 24.6, 13.3, 21.9, and 15.6 IU/L, respectively. More importantly, the increased level of p53 and p21 in HG-stimulated HRECs was repressed by Liraglutide and reversed by lentiviral SIRT1 shRNA (Fig. 8D). These data reveal that the protective effects of Liraglutide in cellular senescence against HG were abolished by the knockdown of SIRT1.

## Discussion

Progressive changes in the retinal microvascular system in diabetic patients are the main features of DR. As a specific mitogen and chemokine of vascular endothelial cells, VEGF promotes the division of vascular endothelial cells and induces cell proliferation, resulting in angiogenesis, which further contributes to the development of DR [25]. Studies have shown that the level of VEGF in the serum of DR patients gradually increases with the aggravation of the disease, suggesting that VEGF plays a vital role in the occurrence of DR and is closely associated with the severity of the disease [26]. Moreover, it is reported that factors secreted by retinal cells, such as IL-6, TNF-α, and TGF-β, stimulate the proliferation of various retinal cell components, leading to the formation of new blood vessels, and thus the occurrence and development of DR [27]. The present study established an in vitro model in HRECs with 30 mM glucose and the model was verified by the extensive release of VEGF and IL-6, which was consistent with the results shown by Zhou [28]. After treatment with Liraglutide, the release of VEGF and IL-6 was greatly repressed, implying a potential inhibitory effect of Liraglutide against the development of DR.

A limited number of proliferations is observed in normal cells. After a certain number of proliferations, a permanent non-dividing state with a specific phenotype is developed in cells, which is named “replicative senescence” [29]. Studies have shown that senescence is mainly caused by telomere shortening and dysfunction as cells continue to divide [30] and is characterized by decreased telomerase activity, upregulated β-galactosidase, and activated p53/p21 axis [31]. Several studies have shown that cell senescence is involved in a variety of vascular-related pathological processes, including DR [32, 33]. We found that in HG-stimulated HRECs, inhibited telomere activity, elevated percentage of SA-β-gal staining positive cells, and activated p53/p21 pathway were observed, which is consistent with the HG-induced cellular senescence reported by Zhou [28]. Cellular senescence in HG-stimulated HRECs was extremely alleviated after Liraglutide incubation, suggesting that the protective effect of Liraglutide on HG-induced injury in HRECs might be associated with the ameliorated cellular senescence.

SIRT1 is the type III histone deacetylase (HDAC) of nicotinamide adenine dinucleotide (NAD) and is a member of the Sirtuins family [24]. Mortuza et al. [34] showed that SIRT1 regulated ET-1 and TGF-β1 through transcriptional coactivator P300, thereby preventing the upregulation of collagen Iα and reducing endothelial cell permeability induced by HG. The elevated expression level of HMGB1 in diabetic retinas is reported to be repressed by the activation of SIRT1, thereby alleviating the retinal endothelial barrier dysfunction caused by diabetes [35]. Recent studies have shown that SIRT1, as an important regulator of cellular senescence [36], is involved in the regulation of a variety of diseases, including DR [37]. Consistent with previous reports ^38,39^, SIRT1 was found to be extremely downregulated in HG-stimulated HRECs. After the incubation of Liraglutide, the expression level of SIRT1 was greatly rescued, suggesting that the function of Liraglutide might be mediated by SIRT1. A further verification study revealed that the protective effects of Liraglutide in cellular senescence against HG were abolished by the knockdown of SIRT1, implying that Liraglutide exerted the anti-senescent property in HG-stimulated HRECs by activating SIRT1. However, the therapeutic effect of Liraglutide on DR will be further identified in our future work using a DR animal model, in which the mechanism of SIRT1 will be further verified.

Collectively, our data reveal that Liraglutide alleviated cellular senescence in HG-stimulated HRECs by activating SIRT1.

## Declarations

### Data availability

The data that support the findings of this study are available from the corresponding author upon reasonable request.
